# Cortactin Promotes Effective AGS Cell Scattering by *Helicobacter pylori* CagA, but Not Cellular Vacuolization and Apoptosis Induced by the Vacuolating Cytotoxin VacA

**DOI:** 10.3390/pathogens11010003

**Published:** 2021-12-21

**Authors:** Irshad Sharafutdinov, Jakob Knorr, Delara Soltan Esmaeili, Steffen Backert, Nicole Tegtmeyer

**Affiliations:** Department of Biology, Division of Microbiology, University of Erlangen-Nuremberg, 91058 Erlangen, Germany; Jakob.Knorr@fau.de (J.K.); delara.esmaeili@fau.de (D.S.E.); Steffen.Backert@fau.de (S.B.)

**Keywords:** *H. pylori*, cortactin, apoptosis, annexin V, caspase-3, cell scattering, CagA, CagE, VacA, FlaA, T4SS

## Abstract

Cortactin is an actin-binding protein and actin-nucleation promoting factor regulating cytoskeletal rearrangements in eukaryotes. *Helicobacter pylori* is a gastric pathogen that exploits cortactin to its own benefit. During infection of gastric epithelial cells, *H. pylori* hijacks multiple cellular signaling pathways, leading to the disruption of key cell functions. Two bacterial virulence factors play important roles in this scenario, the vacuolating cytotoxin VacA and the translocated effector protein CagA of the *cag* type IV secretion system (T4SS). Specifically, by overruling the phosphorylation status of cortactin, *H. pylori* alternates the activity of molecular interaction partners of this important protein, thereby manipulating the performance of cytoskeletal rearrangements, endosomal trafficking and cell movement. Based on shRNA knockdown and other studies, it was previously reported that VacA utilizes cortactin for its cellular uptake, intracellular travel and induction of apoptosis by a mitochondria-dependent mechanism, while CagA induces cell scattering, motility and elongation. To investigate the role of cortactin in these phenotypes in more detail, we produced a complete knockout mutant of cortactin in the gastric adenocarcinoma cell line AGS by CRISPR-Cas9. These cells were infected with *H. pylori* wild-type or various isogenic mutant strains. Unexpectedly, cortactin deficiency did not prevent the uptake and formation of VacA-dependent vacuoles, nor the induction of apoptosis by internalized VacA, while the induction of T4SS- and CagA-dependent AGS cell movement and elongation were strongly reduced. Thus, we provide evidence that cortactin is required for the function of internalized CagA, but not VacA.

## 1. Introduction

*Helicobacter pylori* represents one of the most successful pathogenic bacteria in humans; about half of the world’s population is infected by this microbe [1]. Though in most cases patients remain asymptomatic, prolonged infection with the bacterium has been associated with various gastric diseases such as chronic inflammation of the stomach, peptic ulcers, or gastric adenocarcinoma in a subset of individuals [2,3,4,5]. Because of the capability to induce the development of stomach cancer, *H. pylori* has been classified as the first class I bacterial carcinogen in history [6]. Persistent colonization of the stomach is the main goal of *H. pylori* and proceeds by highly complex and multistep processes. To survive under the harsh conditions in the human stomach, the pathogen neutralizes the surrounding stomach acid to a survivable level by secreting the urease enzyme [7]. To find and penetrate the gastric mucus layer, *H. pylori* can sense differences in the pH gradient of the stomach [8,9]. Once *H. pylori* has reached the gastric epithelial cell surface, it employs a multitude of virulence factors, such as the outer membrane adhesins HopQ, BabA, or SabA to adhere to the cells [10,11,12], or the protease HtrA to disrupt tight and adherent junctions in the gastric epithelium, and gain access to the intercellular space via cleavage of E-cadherin, occludin and claudin-8 to achieve long-term infection [13,14,15]. Two of the best-studied virulence factors are the cytotoxin-associated gene A (*cagA*) and the vacuolating cytotoxin A (*vacA*) [3,4,5]. CagA is encoded by the so-called *cag* pathogenicity island (*cag*PAI), along with a multitude of other proteins (CagY, CagL, CagE, etc.), which form a type IV secretion system (T4SS) to deliver CagA in the host cell [15,16,17,18]. The presence of CagA shows a high correlation with a subset of highly virulent *H. pylori* strains and exhibits a higher risk to develop stomach cancer compared to CagA-negative isolates [19]. After injection by the T4SS, CagA becomes tyrosine-phosphorylated by members of the Src and Abl family of kinases [20,21,22] and can interact with a plethora of different host cell proteins, both in a phosphorylation-dependent and -independent manner to manipulate host cell signaling [23,24]. In the gastric cancer cell line AGS, a very distinctive elongation phenotype can be observed following infection with CagA-positive *H. pylori*, which is characterized by the induction of cell scattering, motility and elongation [25]. In addition, translocated CagA has been shown to trigger anti-apoptosis in the gastric pits by increasing the levels of the pro-survival factor phospho-ERK1/2 (extracellular regulated kinase 1/2) and induction of the anti-apoptotic protein MCL1 [26]. On the other hand, VacA is a pore-forming toxin, which disturbs the cellular integrity of host cells by forming anion-selective channels in the plasma membrane [3,4,5,27]. The *vacA* gene can be found in virtually all strains isolated from human patients, though not all *vacA* alleles exhibit cytotoxic or vacuolating activity [3,4,5,28,29]. VacA of the *s1m1* allelic genotype exhibits the highest known toxic activity and has been associated with the development of gastric diseases [3,4,5,30,31]. Furthermore, internalized VacA can induce the formation of large vacuoles in infected epithelial cells, can travel to the mitochondria and is capable of inducing mitochondria-dependent apoptosis [32,33,34]. Interestingly, manipulation of host cell signaling by CagA and VacA often appears antagonistic; this interaction between the two virulence factors may allow for the bacteria’s fine-tuning of their respective activities [27,35,36]. One of the host cell proteins that has been previously associated both with CagA [37] and VacA [38] activities is the actin-binding protein cortactin. Cortactin is encoded by the *cttn* gene and acts as a stabilizing factor of branched actin networks by binding to the actin nucleation factor Arp2/3 [39,40,41]. Cortactin consists of four major functional domains: an N-terminal acidic domain (NTA), a filamentous actin (F-actin) binding region, a proline-rich domain, and a C-terminal Src homology 3 (SH3) domain [42,43]. The NTA and SH3 domains provide cortactin with interactions by other cytoskeleton regulating proteins, such as neural Wiskott–Aldrich syndrome protein (N-WASP) [44], tight junction protein zonula occludens (ZO)-1 [45], WASP-interacting protein (WIP) [46], focal adhesion kinase (FAK) [37] and many others [40], therefore efficiently regulating cell dynamics. Since cortactin is a key regulator of the cellular actin cytoskeletal organization and cell movement, it is utilized as a major host target during *H. pylori* infection [47]. In particular, *H. pylori* manipulates cortactin’s serine and tyrosine phosphorylation status in a CagA-dependent manner, via activation of the serine kinase ERK1/2 and an as of yet unknown tyrosine phosphatase [37,48]. Serine-phosphorylated cortactin then triggers its binding to FAK and FAK auto-phosphorylation, thereby promoting FAK-dependent signaling and phosphorylation of CagA itself via members of the Src and Abl family of tyrosine kinases [37,49]. Finally, the upregulation of FAK activity prevents excessive gastric epithelial cell lifting, which supports sustained *H. pylori* infections. Taking into account that CagA and VacA have been shown to influence each other’s activity [27,35], it was proposed that cortactin might also interfere with VacA functions during infection. For example, it was reported that treatment with acid-activated VacA resulted in an increased apoptosis of AGS cells overexpressing cortactin, while shRNA knockdown of cortactin reduced the apoptosis rates [38]. Besides, expression of the apoptosis regulating proteins Bcl-2 and Bax were also described to depend on cortactin in AGS cells treated with purified VacA [38]. The intracellular trafficking of VacA in cultivated host cells was shown to associate with actin structures [50] and therefore might also be regulated through cortactin activity. Thus, cortactin was proposed to play a crucial role in the VacA-induced apoptosis, probably by promoting endocytic travel of VacA within the host cell [38]. In the present study, we aimed to take a closer look at the role of cortactin during infection with *H. pylori*, with specific interest regarding the functions of CagA and VacA. To investigate cortactin activity in more detail, we have recently established a stable cortactin knockout in the AGS gastric epithelial cell line using the CRISPR-Cas9 gene-editing tool [49]. Deletion of the *cttn* gene in AGS cells disrupted *H. pylori*-induced signaling, in particular, by diminishing the activation levels of FAK, Src and Abl tyrosine kinases. Furthermore, cortactin was found to be vital in the CagA phosphorylation process [49]. For this purpose, we infected AGS wild-type (wt) and AGSΔ*cttn* knockout cells with *H. pylori* wt and isogenic mutants. We show that cortactin deficiency did not negatively affect the uptake or intracellular function of VacA during infection with *H. pylori*, or by addition of the purified protein, but did diminish the expression of the CagA-dependent elongation phenotype in AGS cells. The VacA-dependent apoptosis and vacuolization rates during *H. pylori* infection or treatments with purified VacA were found to be at similar rates in both the wt and cortactin-deficient AGS cells. Taken together, the presented data shows that cortactin is rather involved in CagA-mediated AGS cell scattering signaling, while its role in VacA-mediated vacuolization and apoptosis seems to be dispensable.

## 2. Results

### 2.1. Cortactin Knockout Is Associated with Diminished AGS Cell Scattering and Elongation Phenotype

**Figure 1:**
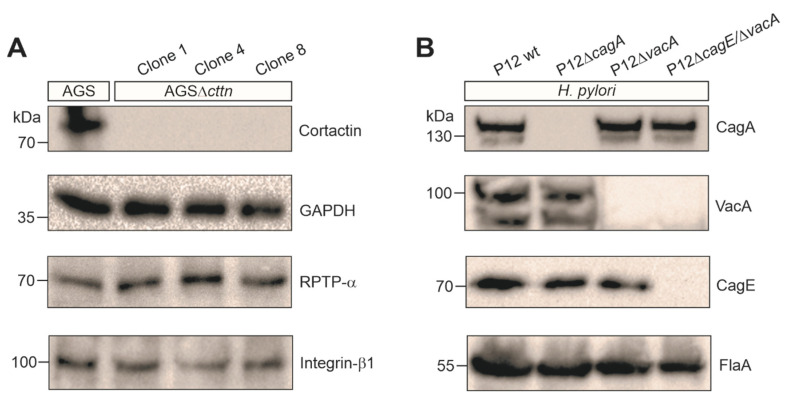
Confirmation of complete gene knockouts in AGS cells and *H. pylori* strains as used in this study. (**A**) Western blot analysis of cortactin deletion in three representative AGSΔ*cttn* clones 1, 4, and 8. The GAPDH blot served as a loading control. Expression of the VacA receptor RPTP-α [52] and integrin-β1 expression, which is required for the AGS cell elongation phenotype [53], were not compromised in the AGSΔ*cttn* knockout cells. (**B**) Confirmed knockout of *cagA*, *vacA* and *cagE* genes in the *H. pylori* mutant strains using specific antibodies against the corresponding proteins. The FlaA blot was used as a loading control.

**Figure 2:**
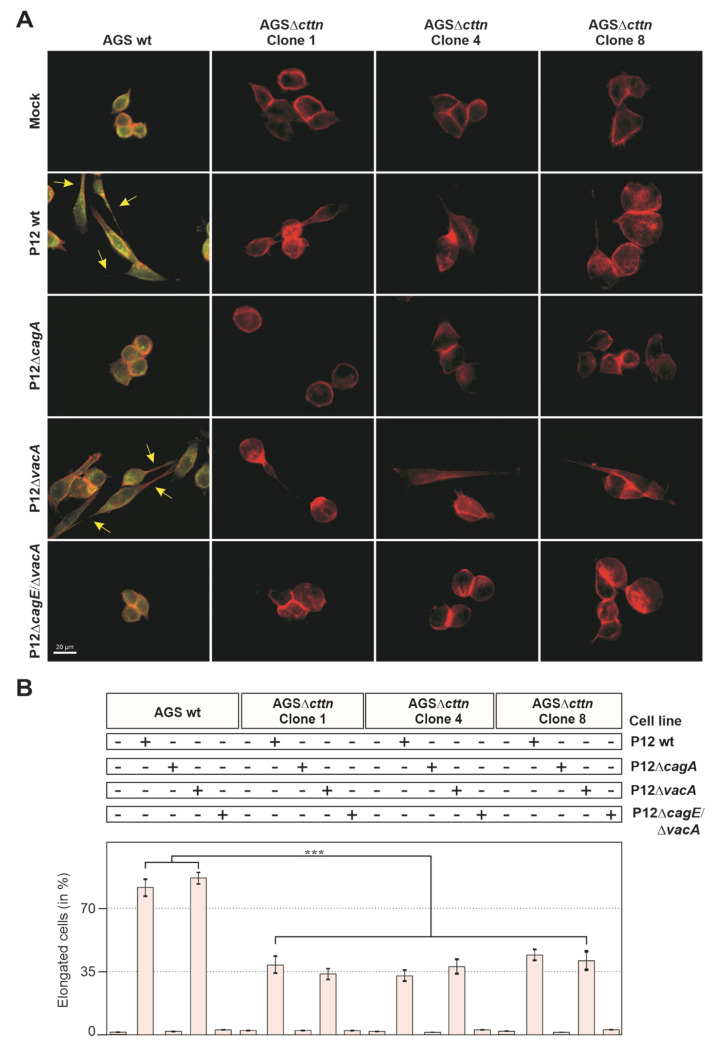
*H. pylori* induces strong CagA-dependent cell elongation in AGS wt cells, while in AGSΔ*cttn* cells this effect is strongly diminished. (**A**) AGS wt and AGSΔ*cttn* cells were infected for 8 h with *H. pylori* P12 wt or its isogenic P12Δ*cagA*, P12Δ*vacA* and P12Δ*cagE*/Δ*vacA* mutants at MOI of 100. The CagA-induced AGS cell elongation phenotype was observed under fluorescence microscopy in cells stained for cortactin (green) and filamentous actin (red). (**B**) The percentage of cells exhibiting the phenotype was quantified; mean values ± standard error are shown with significant difference *** corresponding to *p* ≤ 0.001.

Scattering and elongation, as well as endocytic travelling and apoptosis, are affected in AGS gastric epithelial cells infected by *H. pylori* [22,27,51]. To study whether cortactin deficiency might impact these AGS cell responses during *H. pylori* infection, we utilized the AGSΔ*cttn* knockout cell line that we had previously created using the CRISPR-Cas9 approach [49]. We confirmed the knockout of cortactin in three AGSΔ*cttn* cell clones, termed clone 1, 4 and 8 (Figure 1A). We have chosen the well-described duodenal ulcer *H. pylori* strain P12, which has a functional T4SS and the active *s1/m1 vacA* allel [10,14,15,17,21,37,48,49], and produced isogenic knockouts in the *cagA* and *vacA* genes, as well as a structural T4SS-inactive *cagE* mutant, which is unable to deliver CagA, with additional *vacA* mutation. The correct expression or absence of the resulting proteins were confirmed by Western blotting using *H. pylori*-specific antibodies (Figure 1B). We then infected AGS wt and three AGSΔ*cttn* clones with P12 wt or its isogenic P12Δ*cagA*, P12Δ*vacA* and P12Δ*cagE*/Δ*vacA* mutants. After 8 h of infection, we immunostained AGS cells with specific α-cortactin antibodies, counterstained filamentous actin with TRITC-conjugated phalloidin, and then analyzed the samples by fluorescence microscopy. Infection with P12 wt and P12Δ*vacA* resulted in strong expression of the elongation phenotype in AGS wt cells as expected, while in all AGSΔ*cttn* clones, the number of elongated cells was reduced (Figure 2A). Almost no elongated cells could be found in the non-infected mock control or infections using the isogenic P12Δ*cagA* or P12Δ*cagE*/Δ*vacA* mutants in either AGS wt or AGSΔ*cttn* cells. Quantification analysis revealed that the expression of the elongation phenotype in AGSΔ*cttn* cells, though not completely abolished, was significantly downregulated in infections with both P12 wt and P12Δ*vacA*. P12 wt infection revealed a decrease from approximately 80 ± 5% in AGS wt by roughly half in AGSΔ*cttn* cells (38 ± 4%, 33 ± 3%, and 44 ± 3% for the clones 1, 4, and 8, respectively), which held true for P12Δ*vacA* (85 ± 3% in AGS wt to 33 ± 3%, 38 ± 4% and 42 ± 4% in the clones 1, 4, and 8, respectively) (Figure 2B). Therefore, cortactin appears to be a major signal transducer of *H. pylori*-induced and CagA-dependent AGS cell scattering and elongation.

### 2.2. VacA-Dependent Vacuole Formation Is Not Hampered in Infected AGSΔcttn Cells

**Figure 3:**
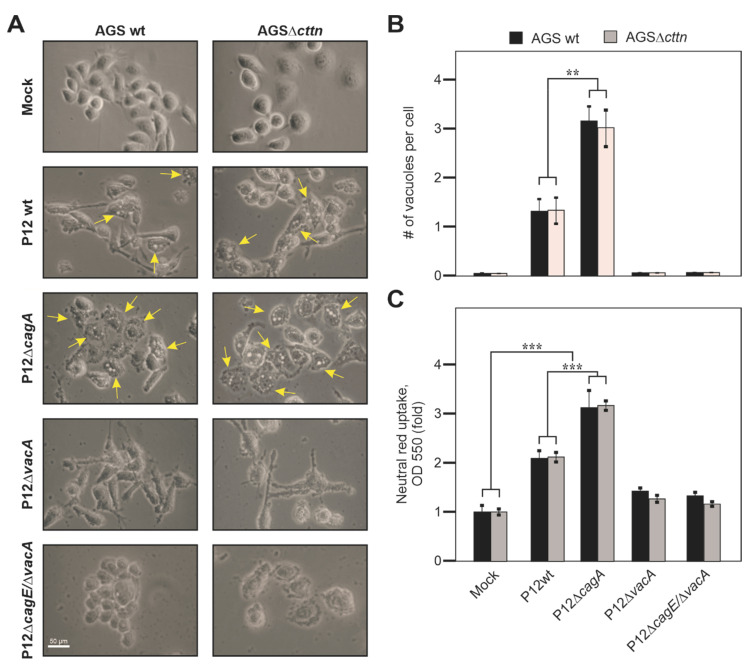
Cortactin is dispensable for VacA-induced cell vacuolization. AGS wt and AGSΔ*cttn* cells were infected with *H. pylori* P12 wt or its isogenic P12Δ*cagA*, P12Δ*vacA* and P12Δ*cagE*/Δ*vacA* mutants at MOI of 100. (**A**) After infection, AGS cells were analyzed using phase contrast microscopy. Vacuolization in AGS cells was assessed by counting the number of vacuoles per cell after 8 h of infection (**B**) and by neutral red uptake assay after 24 h of infection (**C**). The background signal in the neutral red uptake assay of mock control was set as 1. Mean values ± standard error are shown in graphs with significant differences corresponding to *p* ≤ 0.01 (**) and *p* ≤ 0.001 (***).

Besides contributing to CagA signaling, cortactin knockdown by shRNA has been previously reported to be implicated in VacA-dependent activities in AGS cells, when incubated with acid-activated VacA [38]. Therefore, we aimed to identify whether the VacA uptake and its intracellular signaling were affected by the complete lack of cortactin during infection with *H. pylori*. In this experiment, AGS wt exhibited substantial elongation phenotype upon infection with P12 wt and P12Δ*vacA*, which was diminished in AGSΔ*cttn* cells, as seen above. Surprisingly, only minor differences in VacA-dependent vacuole formation were observed in infections regardless of the chosen AGS cell line. Infection with P12 wt resulted in the mild formation of VacA-dependent vacuoles in both AGS wt and AGSΔ*cttn* cells. In contrast, infection with P12Δ*cagA* exhibited stronger vacuole formation, while infections using the P12Δ*vacA* or P12Δ*cagE*/Δ*vacA* mutants displayed no vacuole formation at all, as expected (Figure 3A, example vacuoles indicated by arrows). As assessed by phase contrast microscopy analysis of at least 100 randomly selected cells per experiment, infection with P12 wt resulted on average in the formation of 1.3 and 1.1 vacuoles per AGS wt and AGSΔ*cttn* cell, respectively (Figure 3B). In turn, when infected with the P12Δ*cagA* strain, AGS wt and AGSΔ*cttn* cells revealed on average 2.9 and 3.1 vacuoles, respectively. Therefore, different *H. pylori* mutants revealed varying vacuolating activities, which, however, were independent of the chosen cell line, AGS wt or AGSΔ*cttn*, respectively.

We further analyzed vacuole formation in our AGS cell lines upon *H. pylori* infection by a second approach, the neutral red uptake assay. Similarly, after 24 h of infection, AGS wt and AGSΔ*cttn* cells showed a highly significant increase in vacuolization when infected with P12 wt, and especially P12Δ*cagA*, but not by P12*ΔvacA* or P12*ΔcagE*/Δ*vacA* (Figure 3C). Thus, in concordance with the results obtained by phase contrast microscopy (Figure 3B), the neutral red uptake assay also showed no significant difference in vacuolization between infected AGS wt and AGSΔ*cttn* cells.

**Figure 4:**
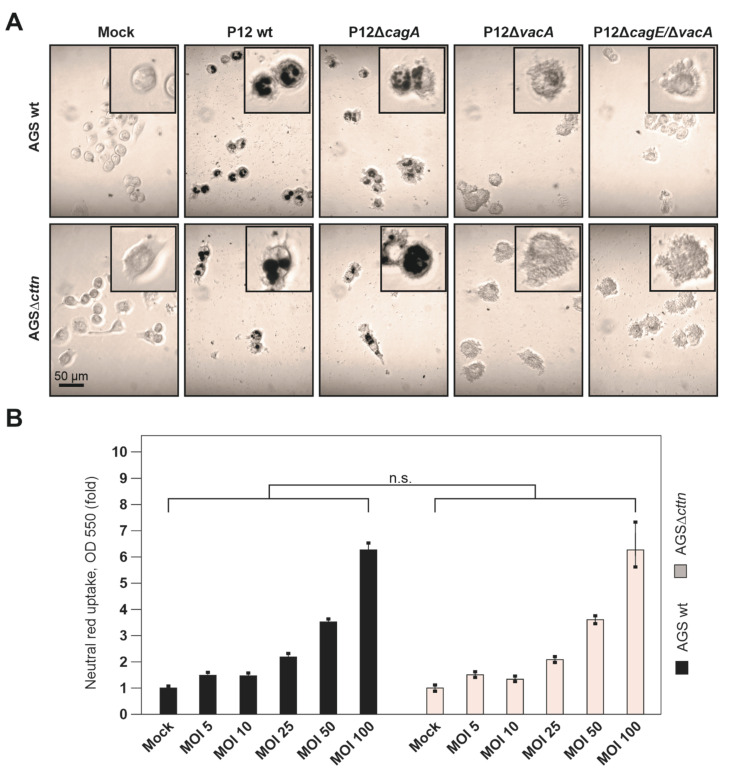
Dose-dependent vacuole formation analyzed by neutral red uptake assay of *H. pylori* infected AGS wt and AGSΔ*cttn* cells. (**A**) Light microscopy with polarizing contrast of AGS wt and AGSΔ*cttn* cells infected with P12 wt or its isogenic P12Δ*cagA*, P12Δ*vacA* and P12Δ*cagE*/Δ*vacA* mutants at MOI of 50 for 24 h, followed by neutral red staining. Vacuoles stained with neutral red were coloured with black. Boxes in the upper corners show representative enlarged AGS cells. (**B**) Additionally, AGS cells were infected with P12 wt in a dose-dependent manner at MOIs of 5, 10, 25, 50 and 100 or were left untreated. After 24 h infection, cells were subjected to neutral red uptake assay, followed by quantification of absorbance intensity. Mean values ± standard error are shown in graphs; n.s., non-significant.

Next, we wanted to analyze if dose-dependent properties could affect vacuole formation during infection. One might take into consideration that the saturation of host cells with VacA due to high bacterial MOIs could result in similar vacuolization quantities between the different AGS cell types. For this purpose, we first infected AGS wt and AGSΔ*cttn* cells with the various *H. pylori* strains for 24 h at MOI of 50, followed by neutral red uptake assay and polarized light microscopy. Similar to our above results for shorter infection times (8 h) and MOI of 100, robust vacuole formation was observed in both AGS cell lines (Figure 4A). To investigate this in more detail, we next infected AGS wt and AGSΔ*cttn* cells with P12 wt in parallel at MOIs of 0, 5, 10, 25, 50, and 100. After 24 h, we performed the neutral red uptake assay and normalized the absorbance data to the number of adhered cells, since an increase in MOI normally led to increased cell lifting over time. However, both AGS wt and AGSΔ*cttn* showed increased vacuolization rates with increasing MOIs, but no significant differences were seen between the two cell lines (Figure 4B).

### 2.3. Cortactin Deficiency Does Not Impact Induction of Apoptosis Provoked by H. pylori

**Figure 5:**
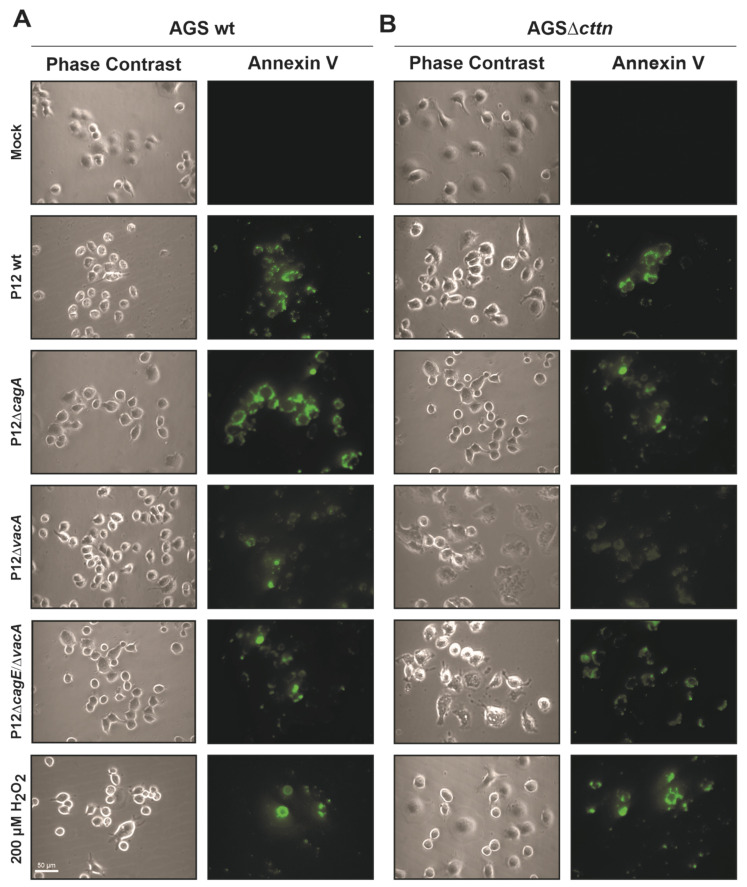
Annexin V staining (green) reveals comparable apoptosis signals between AGS wt and AGSΔ*cttn* cells after infection with *H. pylori*. After 24 h infection with *H. pylori* P12 wt or its isogenic mutants at MOI of 50, AGS wt (**A**) and AGSΔ*cttn* (**B**) cells were analyzed by phase contrast microscopy and fluorescence microscopy. Representative sections on the cover slips are shown.

**Figure 6:**
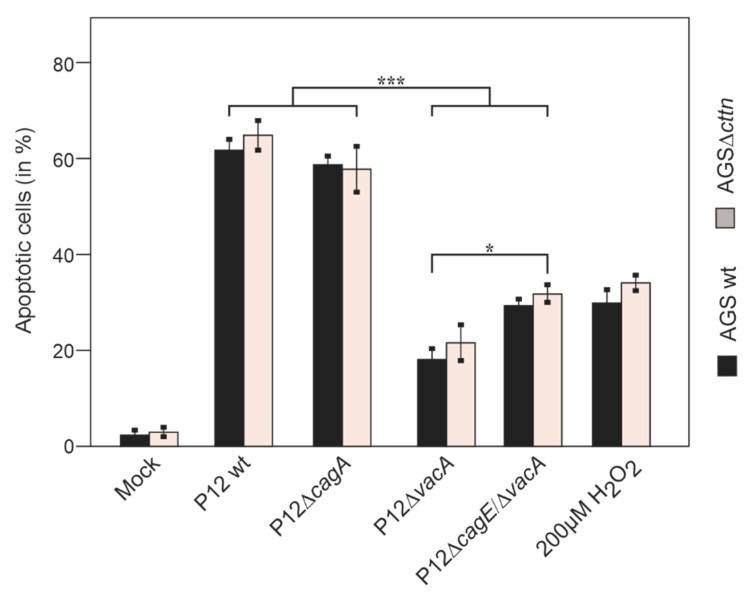
Quantification of apoptosis in AGS wt and AGSΔ*cttn* cells 24 h post-infection. The number of AGS cells stained with Annexin V in Figure 5 was assessed and the percentage of apoptotic cells was determined in three independent experiments. Mean values ± standard error are shown in the graph with significant differences corresponding to *p* ≤ 0.05 (*) and *p* ≤ 0.001 (***).

Since we observed that cortactin apparently had no influence on VacA’s ability to induce cellular vacuolization, we next wanted to take a closer look regarding its role in apoptosis. VacA localizes intracellularly to the membrane of mitochondria, where it induces the release of cytochrome-c into the cytoplasm and thereby induces an apoptotic cascade [33]. To study whether the absence of cortactin affects the induction of apoptosis in AGS cells by *H. pylori*, we infected the cells for 24 h. Addition of 200 μM of H_2_O_2_ to the cells served as a positive control to trigger apoptosis [54]. After infection, the AGS cell lines were stained with the apoptosis marker Annexin V tagged with fluorescein isothiocyanate (FITC), followed by fluorescence microscopy (Figure 5). Quantification of these data is shown in Figure 6.

Interestingly, statistical analysis of the Annexin V staining revealed that the P12Δ*cagA* mutant exhibited only a non-significant difference in apoptosis compared to P12 wt infections. In contrast, infection with the P12Δ*vacA* mutant resulted in reduced apoptosis, as expected. Intriguingly, the double mutant P12Δ*cagE*/Δ*vacA* displayed a slight, but significant increase in apoptosis rate compared to P12Δ*vacA* infection (Figure 6). Thus, it appears that the lack of a functional T4SS and VacA in the P12Δ*cagE*/Δ*vacA* double mutant exhibit anti-apoptotic properties.

**Figure 7:**
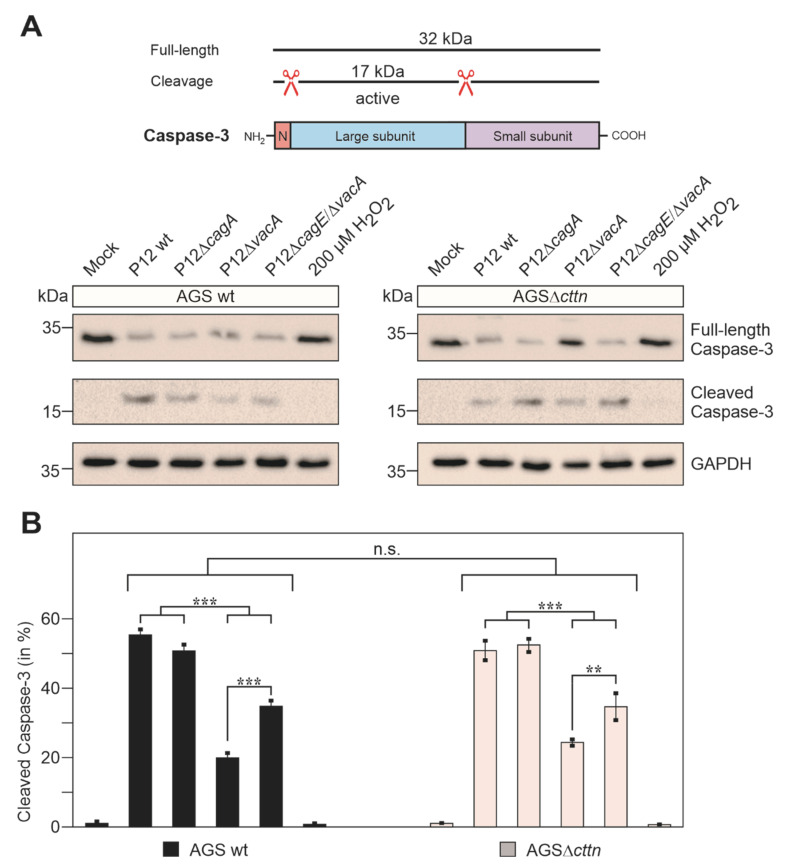
Quantification of caspase-3 cleavage in AGS wt and AGSΔ*cttn* cells 24 h post-infection. (**A**) Schematic representation of caspase-3 domain structure and auto-cleavage events. Full-length caspase-3 is inactive and auto-cleavage between the N-terminal pro-domain and the small C-terminal subunit is necessary for activation of the protein [56]. Western blot analysis shows cleavage of caspase-3 to different extent both in AGS wt and infected AGSΔ*cttn* cells infected at MOI of 50. GAPDH was used as a loading control. (**B**) Quantification of the band intensities of cleaved (active) compared to the total amount of caspase-3 in each sample. Mean values ± standard error are shown in the graphs with significant differences corresponding to *p* ≤ 0.01 (**) and *p* ≤ 0.001 (***).

To corroborate the above findings further, we analyzed another apoptosis marker during infection. It was previously reported that *H. pylori* infection activates caspase-3 to trigger apoptosis [55]. Full-length caspase-3 is approximately 32 kDa in size and, upon activation, two auto-cleavage events release the active 17 kDa large subunit (Figure 7A). Using two commercial α-caspase-3 antibodies recognizing each form, we found that full-length caspase-3 is expressed in the uninfected mock controls of AGS wt and AGSΔ*cttn* cells at similar level, while the active form is not, as expected. Upon infection with P12 wt or P12Δ*cagA*, the intensity of full-length caspase-3 band decreased substantially, and the active subunit band appeared accordingly at the same time (Figure 7A). Thus, these two strains exhibited the strongest capacity to induce apoptosis. In agreement with the Annexin V staining assay described above, the P12Δ*vacA* and P12Δ*cagE*/Δ*vacA* mutants revealed significantly reduced band intensities for the 17 kDa active form and lower rates of apoptosis. Interestingly, no caspase-3 activation at all was observed in the H_2_O_2_-treated positive control, suggesting that H_2_O_2_ might trigger a caspase-3-independent apoptosis pathway. All quantification data are presented in Figure 7B and show similar rates of apoptosis induction in both infected AGS wt and AGSΔ*cttn* cells.

**Figure 8:**
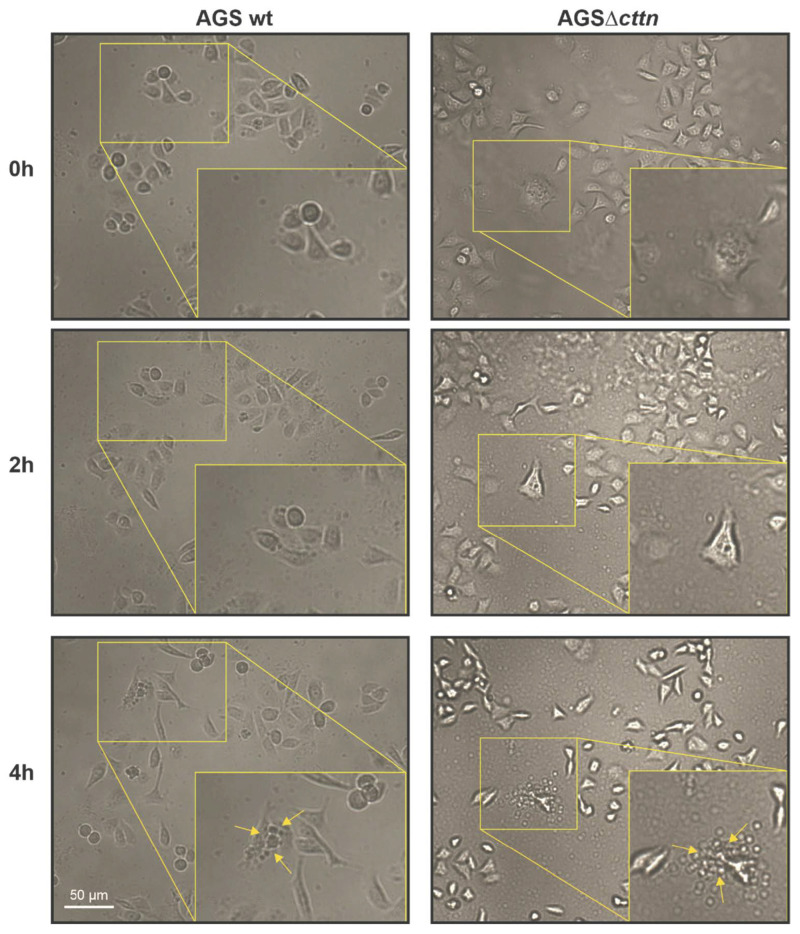
Live cell imaging of AGS wt and AGSΔ*cttn* cells undergoing apoptosis following infection with *H. pylori* at MOI of 50 in a time course. Boxes show enlarged sections with apoptotic bodies of AGS cells that are marked with yellow arrows (bottom).

Next, we performed live cell imaging to investigate apoptosis events and the elongation phenotype in the infected AGS wt and AGSΔ*cttn* mutant cell lines. As observed above, AGSΔ*cttn* cells displayed less elongation than their wt counterparts (Figure 8). The apoptosis occurred at roughly the same time post-infection (200 min in AGS wt versus 180 min in AGSΔ*cttn* cells) and was associated with the occurrence of typical apoptotic bodies when the infected cells burst (Figure 8, arrows). Thus, the knockout of cortactin has no impact on VacA-induced apoptosis in *H. pylori-*infected AGS cells.

### 2.4. Vacuole Formation and Apoptosis by Purified VacA Is Not Affected in AGSΔcttn Cells

**Figure 9:**
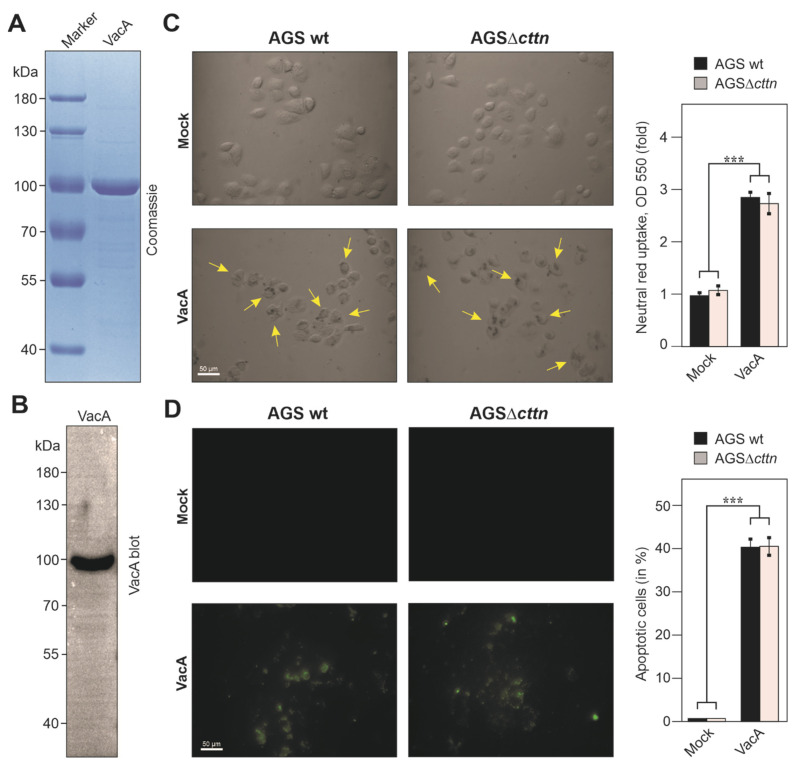
Vacuolization and apoptosis in AGS wt and AGSΔ*cttn* cells was induced by purified acid-activated VacA after 24 h of co-incubation. (**A**) Coomassie-stained purified VacA fraction. (**B**) Western blot analysis of purified VacA. (**C**) Light microscopy with polarizing contrast of AGS wt and AGSΔ*cttn* cells. Vacuoles were stained with neutral red, which is visualized in black (yellow arrows). Neutral red uptake was quantified via absorbance measurements. (**D**) Immunofluorescence microscopy of AGS wt and AGSΔ*cttn* cells. Apoptotic cells were stained using Annexin V. The rates of apoptosis were quantified in triplicate and presented as percentage of total number of cells. Representative sections of the slides are shown. Mean values ± standard error are shown in the graphs with significant differences corresponding to *p* ≤ 0.001 (***).

It was previously reported that treatment of AGS wt cells with purified acid-activated VacA alone induced apoptosis, while shRNA knockdown of cortactin significantly reduced the apoptosis rates [38]. Because our above results from infection experiments are in contrast to this study, we purified and acid-activated VacA, followed by treatment of AGS wt and AGSΔ*cttn* cells using the same amounts under the same conditions, as previously described [38]. Our purified VacA fraction was checked by Coomassie brilliant blue staining and confirmed via Western blot using a specific VacA antibody (Figure 9A,B). However, similar to the infections with live *H. pylori*, the formation of vacuoles was observed and quantified using the neutral red staining method. We observed a highly significant 2.8-fold increase of neutral red uptake in AGS wt cells and a 2.7-fold increase in AGSΔ*cttn* cells. Statistical analysis confirmed that these differences between the cell lines are not significant (Figure 9C). Next, we investigated the induction of apoptosis by purified acid-activated VacA. Similar to the vacuolization assay, we found no significant differences between the two cell lines in regard to the induction of apoptosis (Figure 9D). Therefore, we confirmed that the effect of purified acid-activated VacA both on AGS wt and AGSΔ*cttn* cells is not dependent on the expression of cortactin.

## 3. Discussion

Cortactin, with its important function in the actin-cytoskeletal organization, has been identified as a prominent target of various pathogenic microbes such as *Shigella flexneri* [57], *Cryptosporidium parvum* [58], *Helicobacter pylori* [37], *Trypanosoma cruzi* [59] and others [42]. In fact, cortactin represents such an attractive target protein to hijack the actin cytoskeleton that it has therefore been called an “Achilles’ heel” of the host cell [42]. Cortactin functions as an interaction partner of multiple signaling proteins via its SH3 domain, enabling it to influence various cellular properties such as cell-to-cell junctions [45], migration [60], or endocytosis [61]. Therefore, it comes as no surprise that *H. pylori* is also capable to manipulate the host cell via cortactin. Specifically, translocated CagA triggers the tyrosine dephosphorylation of cortactin via a yet unknown phosphatase [26,48] and induces the serine phosphorylation of cortactin [37] through activation of the ERK1/2 kinase signaling pathway [37,62]. Afterwards, serine-phosphorylated cortactin induces the auto-phosphorylation of FAK at tyrosine 397, which triggers a signaling pathway involving the activation of Src and Abl kinases, which results in the pronounced phosphorylation of injected CagA [49]. Furthermore, it was shown that *H. pylori* triggers cortactin overexpression at the protein level in a CagA- and JNK-dependent manner, which can be associated with gastric carcinogenesis [63]. However, other *H. pylori* virulence factors such as VacA have also been reported to target cortactin. Previously, Chang and co-workers [38] developed DNA constructs to either overexpress or knockdown cortactin in AGS cells. Based on such experiments, in combination with adding purified, acid-activated VacA, they reported that cortactin is involved in the intracellular trafficking and function of VacA [38]. However, similar studies using clean cortactin knockout cells are not yet available.

We performed *H. pylori* infection experiments using AGS wt and AGSΔ*cttn* knockout cells under identical conditions and found that the characteristic phospho-CagA-dependent AGS elongation phenotype was strongly diminished in the cortactin knockout cells. This is in agreement with our previous infection studies, which showed a significant reduction of elongated cells by cortactin siRNA [37] and reduced CagA phosphorylation levels in cortactin-deficient AGS cells compared to control cells [49]. In contrast to the report by Chang and co-workers [38], we, surprisingly, could clearly detect VacA-dependent vacuole formation in AGSΔ*cttn* cells at similar level compared to AGS wt cells, suggesting that the internalization and intracellular function of VacA was not hampered by the complete absence of cortactin. Thus, the intracellular transport of VacA by the endocytic machinery proceeds in a cortactin-independent fashion. In fact, cortactin was shown to be involved in clathrin-mediated endocytosis through direct interaction with the GTPase dynamin [61]. However, there is some evidence that VacA exploits the endocytic pathway independent of clathrin or dynamin [50]. Our observation that VacA’s vacuolating function was not diminished encouraged us to investigate if cortactin deficiency might affect pro- or anti-apoptotic properties of *H. pylori*. For this purpose, we infected AGS wt and AGSΔ*cttn* cells with *H. pylori* wt and isogenic deletion mutants, namely Δ*cagA*, Δ*vacA*, and Δ*cagE*/Δ*vacA* followed by staining with the apoptosis marker Annexin V. In our experiments, AGS cells with the *cttn* gene knockout demonstrated no statistically significant difference in *H. pylori*-induced apoptosis compared to AGS wt cells. In agreement with the previously reported apoptotic activity of VacA [33,34], we found that infection with the P12Δ*vacA* mutant resulted in a significant reduction of the number of apoptotic AGS cells compared to infection with P12 wt. However, the P12Δ*cagE*/Δ*vacA* double mutant induced a slightly increased number of apoptotic cells compared to P12Δ*vacA*, which is in agreement with CagA’s proposed anti-apoptotic properties [26]. In fact, the *cagE* gene of *H. pylori* encodes one of the proteins involved in the structural formation of the T4SS, which injects CagA into the host cell [18]. However, we found only a non-significant difference in the number of apoptotic AGS cells upon infection with the P12Δ*cagA* mutant compared to wt infections. These observations were further confirmed when we assessed the activation of executioner caspase-3. Since the double mutant P12Δ*cagE*/Δ*vacA* gene induced higher apoptosis rates of AGS cells compared to P12Δ*vacA*, we assume that a T4SS effector other than CagA might be the primary anti-apoptotic determinant of *H. pylori.* Injected ADP-Heptose [64] is such a potential candidate. Together, it seems that other *H. pylori* factors might enhance or antagonize the pro-apoptotic function of VacA during infection. However, these results do not explain the differences in the study by Chang and co-workers [38]. We assume that the authors might have seen minor differences, which were over-interpreted in their discussion.

## 4. Material and Methods

### 4.1. Cultivation of Eukaryotic Cells

The AGS cell line derived from the human gastric adenocarcinoma (ATCC CRL-1739) was routinely cultivated at 37 °C and 5% CO_2_ in the RPMI 1640 medium (Gibco, Darmstadt, Germany) [65]. The growth media contained 10% fetal calf serum (FCS, Gibco), 1% penicillin/streptomycin (Sigma-Aldrich, Steinheim, Germany) and 0.2% normocin (InvivoGen, Toulouse, France). AGSΔ*cttn* cells have been generated previously [49] and were grown under the same conditions as AGS wt, except for an additional 2 μg/mL of puromycin in the media. The cells were washed two times with PBS and given fresh media without antibiotics before *H. pylori* infection experiments.

### 4.2. Cultivation of H. pylori Strains

*H. pylori* strain P12 was grown from stock, stored at −80 °C in BHI media with 20% glycerol, onto GC-Agar plates containing 10% horse serum, 10 μg/mL vancomycin and 4 μg/mL amphotericin [66]. Microaerophilic conditions for bacterial growth were provided using CampyGen (Oxoid) gas packages in 2.5 L anaerobic jars (Oxoid, Wesel, Germany). *H. pylori* were grown for approximately 48 h at 37 °C, resuspended in BHI media, replated onto a fresh agar plate and incubated for additional 24 h at 37 °C under microaerophilic conditions before they were used for infection experiments. *H. pylori* deletion mutants P12Δ*cagA*, P12Δ*vacA* and P12Δ*cagE*/Δ*vacA* have been routinely generated by standard gene disruption procedures [67,68], and were grown the same way as the wt strain P12, except for adding 8 μg/mL kanamycin (P12Δ*cagA* and P12Δ*cagE*/Δ*vacA*) or 4 μg/mL chloramphenicol (P12Δ*vacA*) into the agar plates.

### 4.3. Infection of Eukaryotic Cells with H. pylori and Quantification of the Elongation Phenotype

AGS wt and AGSΔ*cttn* cells were grown in 6- or 12-well plates as stated above. Cells were starved overnight in plain RPMI medium, before infection. *H. pylori* were cultivated as indicated above, resuspended in BHI media and added to the AGS cells at a calculated multiplicity of infection (MOI), as indicated in each figure legend. Infected cells were incubated at 37 °C and 5% CO_2_ for 4 h to 24 h depending on the experiment. Quantification of the elongation phenotype was done as described in detail [51]. Briefly, cells were considered to exhibit the elongation phenotype if they displayed thin, needle-like protrusions of a length of 20 μm to 70 μm, as well as a general elongated shape. For quantification purposes, 100 randomly selected cells per experiment were counted and evaluated. The resulting data are from three independent experiments.

### 4.4. SDS-PAGE and Immunoblot Analysis

AGS wt and AGSΔ*cttn* cells intended for SDS-PAGE were harvested with hot (95 °C) SDS-buffer, followed by separation of the proteins in the lysates, according to size via 6–8% polyacrylamide gels. The proteins were then transferred onto a PVDF membrane for Western blotting and probed with antibodies [69]. Prior to probing, the PVDF membranes were blocked with either 3% BSA or 5% non-fat dry milk in TBST, depending on the primary antibody and manufacturers instructions [70]. Primary antibodies used were specific against cortactin (Merck-Millipore, Darmstadt, Germany #05-180), GAPDH (Santa Cruz, Heidelberg, Germany, #SC-47724), CagA (Austral Biologicals, San Ramon, CA, USA, #HPP-5003-9), CagE [63], VacA [34], FlaA [71], RPTP-α (Santa Cruz, #SC-28907), integrin-β1 (Santa Cruz, #SC-H96), full-length caspase-3 (Cell Signaling Technology, Frankfurt, Germany, #9668) and active caspase-3 (Cell Signaling Technology, #9664). Secondary antibodies detected either mouse (Invitrogen, Darmstadt, Germany, #31446) or rabbit (Invitrogen, #31460) primary antibodies and were coupled to horseradish peroxidase. Development of Western blots was performed as previously described [72].

### 4.5. Light and Fluorescence Microscopy

AGS wt and AGSΔ*cttn* cells were grown and infected as described above. After 8 h of infection, the cells were fixed using 4% PFA for 10 min at 20 °C. After fixation, the cells were permeabilized with 0.25% Triton-X-100 for 10 min and stained with mouse α-cortactin antibodies (Merck-Millipore, #05-180) followed by adding the secondary, FITC-conjugated goat-anti-mouse antibody. In addition, cells were counterstained with TRITC-conjugated phalloidin (Thermo Fisher Scientific, Darmstadt, Germany, #R415). Samples were analysed using a Leica DMI4000B fluorescence microscope (Leica Microsystems, Wetzlar, Germany). In order to analyze *H. pylori*-induced vacuoles, infected AGS cells (24 h) were stained with neutral red (see below) and imaged under light microscopy with polarizing contrast. Images were processed using the LAS AF computer software (Leica Microsystems).

### 4.6. Annexin V Apoptosis Assay

AGS wt and AGSΔ*cttn* were grown to approximately 50% confluency and starved overnight in RPMI plain media before being infected with *H. pylori*. Infected cells were incubated for 24 h at 37 °C and 5% CO_2_. Following infection, the cells were washed two times with HEPES-buffer (10 mM HEPES, 140 mM NaCl, 2.5 mM CaCl_2_.) and then incubated at 20 °C in the dark with 0.75 μg/mL Annexin V conjugated with FITC for 30 min. Afterwards, the cells were washed once with HEPES-buffer and were analyzed via fluorescence microscopy. The number of fluorescence positive/apoptotic cells was assessed and the percentage of apoptotic cells compared to the total cell number was determined. Two hundred μM H_2_O_2_ was used as a positive control.

### 4.7. Quantification of Vacuolating Activity

AGS wt and AGSΔ*cttn* cells were grown to 70% confluency in a 6-well plate and infected with *H. pylori* P12 wt, P12Δ*cagA*, P12Δ*vacA* or P12Δ*cagE*/Δ*vacA* and incubated for 8 h at 37 °C and 5% CO_2_. The samples were analyzed by phase contrast microscopy following the infection and the number of formed vacuoles were counted in 100 cells per experiment in triplicate.

### 4.8. Neutral Red Staining

AGS wt and AGSΔ*cttn* cells were grown to approximately 70% confluency and then infected with *H. pylori* strain P12 wt and its isogenic deletion mutants as described above. The infected cells were incubated for 24 h at 37 °C and 5% CO_2_. Following the infections, vacuolization of AGS wt and AGSΔ*cttn* cells was determined via staining of the vacuoles by neutral red (Nutritional Biochemicals Corporation, Cleveland, OH, USA, #12-935). For this purpose, a fresh solution of 0.5% neutral red in PBS with 0.3% BSA was prepared and filtered through a Whatman paper. Cells were washed once in PBS with BSA and then incubated with the neutral red solution for 5 min at 20 °C. Afterwards, the cells were washed again three times with PBS containing 0.3% BSA. Staining of vacuoles was confirmed by light microscopy with polarizing contrast. For quantitative analysis, neutral red was removed from the cells with acidified 70% ethanol and optical density (OD) was measured at 550 nm [73]. Results were calculated as fold change in infected cells compared to mock control. All measurements were performed in triplicate. Results were normalized to the number of adherent cells. The number of adherent cells was determined as the average number of cells in five different 1 mm² squares of each infected well.

### 4.9. Live Cell Imaging

To determine the time of morphological changes and apoptosis AGS wt and AGSΔ*cttn* were grown in 6-well plates to 70% confluency and infected with P12 wt for 4 h at MOI of 50. Images were captured using the JuLI Live Cell Movie Analyzer (NanoEnTek Inc., Waltham, MA, USA) in intervals of 5 min.

### 4.10. In Vitro VacA Preparation and Acid-Activation

VacA was purified from *H. pylori* culture supernatants as described [73] and tested via SDS-PAGE, either stained with Coomassie brilliant blue R-250 (Bio-Rad Laboratories, Feldkirchen, Germany, #161-0400) [74] or Western blot with a specific VacA antibody (see above). Purified VacA protein was acid-activated as described in [38]. In brief, VacA was diluted in RPMI plain medium adjusted to pH = 2 by addition of HCl and NH4Cl and incubated at 37 °C for 15 min. Afterwards the pH was neutralized back to pH = 7 via addition of NaOH. AGS wt and AGSΔ*cttn* cells were co-incubated with a final concentration of 5 ng/mL acid-activated VacA for 24 h. Following co-incubation, neutral red and Annexin V stainings were performed, to quantify vacuolization and apoptosis respectively, as described above.

### 4.11. Statistics

All experiments were performed in triplicate. Relative amounts of caspase-3 were determined via Image Lab Software (Version 6.1; Bio-Rad Laboratories) to measure band intensities densitometrically. Statistical significance was evaluated using one-way ANOVA, followed by Tukey’s test with GraphPad Prism statistical software version 8.0 (GraphPad Software, United States, www.graphpad.com, accessed on 24 November 2021). Statistical significance was defined as *p* ≤ 0.05 (*), *p* ≤ 0.01 (**) and *p* ≤ 0.001 (***).

## Data Availability

The data that support the findings of this study are available from the corresponding author, N.T., upon reasonable request.
